# Fasudil in Combination With Bone Marrow Stromal Cells (BMSCs) Attenuates Alzheimer’s Disease-Related Changes Through the Regulation of the Peripheral Immune System

**DOI:** 10.3389/fnagi.2018.00216

**Published:** 2018-07-16

**Authors:** Jiezhong Yu, Yuqing Yan, Qingfang Gu, Gajendra Kumar, Hongqiang Yu, Yijin Zhao, Chunyun Liu, Ye Gao, Zhi Chai, Jasleen Chumber, Bao-Guo Xiao, Guang-Xian Zhang, Han-Ting Zhang, Yuqiang Jiang, Cun-Gen Ma

**Affiliations:** ^1^Institute of Brain Science, Shanxi Datong University, Datong, China; ^2^State Key Laboratory of Molecular Developmental Biology, Institute of Genetics and Developmental Biology, Chinese Academy of Sciences (CAS), Beijing, China; ^3^Department of Biomedical Sciences, City University of Hong Kong, Kowloon, Hong Kong; ^4^2011 Collaborative Innovation Center, Research Center of Neurobiology, Taiyuan, China; ^5^Departments of Behavioral Medicine and Psychiatry & Physiology, Pharmacology & Neuroscience, The Blanchette Rockefeller Neurosciences Institute, West Virginia University Health Sciences Center, Morgantown, WV, United States; ^6^Institute of Neurology, Huashan Hospital, Institutes of Brain Science and State Key Laboratory of Medical Neurobiology, Fudan University, Shanghai, China; ^7^Department of Neurology, Thomas Jefferson University, Philadelphia, PA, United States

**Keywords:** Alzheimer’s disease, BMSCs, Fasudil, peripheralral immune system, cognition

## Abstract

Alzheimer’s disease (AD) is a chronic progressive neurodegenerative disease. Its mechanism is still not clear. Majority of research focused on the central nervous system (CNS) changes, while few studies emphasize on peripheral immune system modulation. Our study aimed to investigate the regulation of the peripheral immune system and its relationship to the severity of the disease after treatment in an AD model of APPswe/PSEN1dE9 transgenic (APP/PS1 Tg) mice. APP/PS1 Tg mice (8 months old) were treated with the ROCK-II inhibitor 1-(5-isoquinolinesulfonyl)-homo-piperazine (Fasudil) (intraperitoneal (i.p.) injections, 25 mg/kg/day), bone marrow stromal cells (BMSCs; caudal vein injections, 1 × 10^6^ BMSCs /time/mouse), Fasudil combined with BMSCs, or saline (i.p., control) for 2 months. Morris water maze (MWM) test was used to evaluate learning and memory. The mononuclear cells (MNCs) of spleens of APP/PS1 Tg mice were analyzed using flow cytometry for CD4+ T-cells, macrophages, and the pro-inflammatory and anti-inflammatory molecules of the macrophages. Immunohistochemical staining was used to examine the expression of ROCK-II in the spleens of APP/PS1 Tg mice. The MWM test showed improved spatial learning ability in APP/PS1 Tg mice treated with Fasudil or BMSCs alone or in combination, compared to untreated APP/PS1 Tg mice. Fasudil combined with BMSCs intervention significantly promoted the proliferation of CD4+/CD25+ and CD4+/ IL-10 lymphocytes, induced the release of cytokine factors, and regulated the balance of the immune system to work functionally. It also shifted M1 (MHC-II, CD86) to M2 (IL-10, CD206) phenotype of macrophages of CD11b and significantly enhanced the anti-inflammatory and phagocytic abilities (CD16/32) of macrophages of CD11b. Immunohistochemical staining showed significantly decreased expression of ROCK-II in mice treated with combination of Fasudil with BMSCs as compared to saline control. Fasudil in combination of BMSCs improved cognition of APP/PS1 Tg mice through the regulation of the peripheral immune system, including reduction of ROCK-II expression and increased proportion of anti-inflammatory M2 mononuclear phenotype and phagocytic macrophages in the spleen of the peripheral immune system. The latter was achieved through the communication between brain and spleen to improve the immunoregulation of CNS and AD disease conditions.

## Introduction

More than 47 million people worldwide suffer from Alzheimer’s disease (AD) and this number will be increased to 131 million by 2050 (Prince et al., 2016). AD is a progressive neurodegenerative disease, pathologically characterized by amyloid-β (Aβ) peptide plaques and tau protein neurofibrillary tangles. It starts from the hippocampus of the brain, which is an anatomical site for the formation of initial memory. The plaques and tangles from these two proteins destroy neuronal cells of the brain and eventually impair the patient’s memories. Gradually, AD patients lose the ability to control directional leading, causing profound confusion. The mechanisms of the AD process are largely unknown. AD primarily affects the central nervous system (CNS). However, communications between the CNS and peripheral immune system can play an important role in AD. We believe that the mechanism of AD involves immunity dysfunction. There are limited studies on the role of peripheral immune system in AD. Therefore, it was of interest and importance to investigate how the peripheral immune system contributes to the development of AD (Subramanian et al., 2010).

APP/PS1 transgenic (APP/PS1 Tg) mice are widely used in AD studies. APP/PS1 Tg mice start to exhibit impairment of learning and memory at the age of 8 months (Li et al., 2016) and significant plaques in the hippocampus at 9 months (Jankowsky et al., 2004).

Rho-associated protein kinase (ROCK) has been considered as a promising drug target for the prevention of neurodegenerative and neurological diseases (Mueller et al., 2005). ROCK-II plays an important role in majority of nervous diseases, such as multiple sclerosis (MS), spinal cord injury, stroke and AD. We have already shown in our previous studies that 1-(5-isoquinolinesulfonyl)-homo-piperazine (Fasudil), a selective ROCK inhibitor (Xin et al., 2015) decreases expression of ROCK-II in the spleens of experimental autoimmune encephalomyelitis (EAE) C57/BL6 mice (Yu et al., 2010). We also demonstrated that Fasudil limits leukocyte activation and infiltration in several models of inflammation, such as EAE or Parkinson’s disease (PD; Hou et al., 2012; Zhao et al., 2015). In addition, Fasudil protects neurons and mobilizes neural stem cells in mice through conversion of pro-inflammatory M1 macrophage to anti-inflammatory M2 cells type. Its modulation of microglial polarization toward the M2 phenotype may be involved in future therapeutic and preventive strategies for neuroinflammatory and neurodegenerative diseases (Song and Suk, 2017). Human bone marrow stromal/stem cells (BMSCs) have been reported as immunomodulatory functions (Wada et al., 2011). Eftekharzadeh et al. (2015) has demonstrated that BMSCs injections in the rat AD model decrease neurotoxicity of Aβ, indicating that BMSCs could be a potential therapy for AD (Wu et al., 2007). These lines of evidence have offered us a new treatment strategic plan that Fasudil and BMSCs might be promising agents for the prevention and treatment of inflammation-related diseases, such as AD. Since Fasudil and BMSCs have been demonstrated to regulate the immune system, we hypothesized that combination of both have better treatment efficacy in the mouse model of AD.

AD was described for the first time by Alois Alzheimer in 1906 (Mietelska-Porowska and Wojda, 2017), but the mechanisms of AD pathogenesis and progression have not been elucidated to date. It has been shown that regulatory T-cells (Tregs) exert an impact on cognitive function (Baek et al., 2016) via decreased Aβ deposition. Depletion of Tregs accelerates the onset of cognitive deficit and increases Aβ deposition. It is well known that IL-10 modulates immune responses in AD patients (Torres et al., 2013) and TGF-β signaling in T-cells to mandates the induction of immune tolerance (Baas et al., 2016). Hence, neurodegeneration in AD may be contributed by peripheral immune responses (Sil et al., 2016). However, little is known about the changes in the spleen and the association with the peripheral immune system that might contribute to AD pathology (Subramanian et al., 2010). Therefore, present study was aimed to explore the pathology of AD under the influence of peripheral immune system. We used APP/PS1 Tg mice as a model of AD and examined the effects of Fasudil, BMSCs, or the combination of both on cognition, the peripheral immune system, and inflammatory responses. We observed that the combination of Fasudil with BMSCs enhanced memory, activated the peripheral immune reactions, and produced anti-inflammatory effects in AD mice.

## Materials and Methods

All experiments were performed in compliance with the guidelines and regulations of Administration Office of the International Council for Laboratory Animal Science. The experimental protocols were approved by the animal Ethics Committee of Datong University, Shanxi, China. Animals had access to standard food and water *ad libitum*.

### Animals and Drug Treatment

Male APPswe/PSEN1dE9 transgenic (APP/PS1 Tg) mice (8 months old), expressing human amyloid precursor protein (HuAPP695swe) and a mutant human presenilin 1 (PS1-dE9), were purchased from Model Animal Research Center of Nanjing University (Nanjing, China). All animals were housed in pathogen-free animal house facility at the Institute of Brain Science, Datong University and maintained 12/12 light/dark cycle (25 ± 2°C, humidity 50 ± 5%). APP/PS1 Tg mice were screened based on the normal physiological behavior and randomly divided into four treatment groups: (1) Normal control mice (NS; *n* = 9), which were administered normal saline (volume was adjusted similar to Fasudil treatment, i.p., 8 weeks); (2) Fasudil-treated (F, *n* = 8) mice, which received daily i.p. injection of Fasudil (Tianjin Chase Sun Pharmaceutical Co., Ltd.), 25 mg/kg/day, 8 weeks; (3) BMSCs (National Engineering Laboratory for Resource Developing of Endangered Chinese Crude Drugs in Northwest of China, Shanxi Normal University) treated (BM, *n* = 10) mice, which were administered BMSCs (1 × 10^6^ BMSCs per mice) in the caudal vein (intravenous, i.v.), once every 2 weeks for 8 weeks; and (4) combination therapy group, i.e., Fasudil with BMSCs treatment (FBM, *n* = 10), which received Fasudil and BMSCs at the similar dose and duration for 8 weeks. The dose of Fasudil and BMSCs was selected based on our preliminary experiments and clinical dosage (Yang et al., 2010; Gu and Zhang, 2017; Yu et al., 2017).

### Cultures of Bone Marrow Stromal Cells (BMSCs)

BMSCs were isolated and cultured as previously reported (Yu et al., 2017) and described by Jackson Laboratory and National Engineering Laboratory for Resource Developing of Endangered Chinese Crude Drugs in NW of China. Whole bone marrow (BM) was harvested from the femur of female C57BL/6 mice (8–12 weeks old) for BMSCs generation. After isolation, BMSCs were cultured in Neurobasal-A-Medium (Gibco) supplemented with 2% B27 (Gibco), 0.1% Beta-Thiolhistidine (Gibco), 100 U/ml Penicillin and 100 μg/ml Streptomycin, 1% MEM Non-Essential Amino Acids Solution (Gibco), 1% Sodium Pyruvate 100 mM Solution (Gibco), 1% L-Glutamine (Gibco), and Epidermal Growth Factor/Fibroblast Growth Factor. All cells were cultured in an incubator (5% CO_2_ at 37°C). Small neurospheres were observed when BMSCs were cultured to the 5th–10th passages. Neurospheres were gently dissociated to single BMSCs by accutase in our experiments. BMSCs were suspended at 1 × 10^6^ cells in 500 μl of normal saline before injecting into the mouse by tail vein every time.

### Mouse Behavior Tests

The Morris water maze (MWM) test was performed to assess spatial learning and memory ability 2 weeks after the last treatment using a gray plastic round pool (90 cm in diameter). The pool was filled with opaque water maintained at constant temperature (25 + 2°C) and permanent visual cues on the walls of pool with quiet environment and consistent lighting, as previously described (Yu et al., 2017). The MWM was conceptually divided into four quadrants: Northeast (NE), Northwest (NW), Southeast (SE), and Southwest (SW); and eight zones: the Border NW zone, Internal NW zone, Border NE zone, Internal NE zone, Border SW zone, Internal SW zone, Border SE zone, Internal SE zone. In the center of the target quadrant (Internal SW zone), there was a 5.0 × 5.0 cm transparent platform hidden 2.0 cm under the water surface. During the 5-day acquisition training, mice were individually trained to locate the hidden platform from the starting point for two trials/day with the cutoff time at 60 s. Mice were guided to the platform and allowed to stay on the platform for 10 s if it did not find the platform at 60 s. The probe trial was performed on day 7 after the last training trial, during which the platform was removed and mice was allowed to swim freely for 60 s. The position of mice was tracked by a camera above the center of the pool. The camera was connected to an automated video acquisition and analysis system (SMART V3.0 system, Panlab, Barcelona, Spain). Several parameters such as latency to the target (SW) zone, latency of the 1st entrance to the SW zone, mean distance to and time in the SW zone (%), distance traveled in the SW zone (%), and global activity in the SW zone (%) were used for analysis using the SMART V3.0 system. These targets were used to indicate the degree of memory consolidation after training.

### Preparation Mononuclear Cells (MNCs) of Spleen

Mice were sacrificed after completion of the MWM test. Spleens were isolated under aseptic conditions and suspensions of splenic mononuclear cells (MNCs) were prepared by grinding the spleens through a 40 mm nylon mesh in pre-cooled DMEM. Subsequently, red blood cells (RBC) in the suspensions were osmotically lysed. MNCs were washed three times with PBS before resuspending in DMEM with 10% fetal bovine serum.

### Flow Cytometry Analysis

Splenic MNCs were adjusted to 3 × 10^6^/ml and divided into two parts. For cell surface staining, the MNCs were stained for 20 min at RT in 1% BSA-PBS buffer with the following panel of antibodies: FITC-CD4, FITC-CD11b, PE-CD25, PE-CD206, PE-CD86 and PE-CD16/32 (eBioscience, San Diego, CA, USA; 1:1000). For intracellular staining, MNCs were stained for 20 min at RT in 0.3% saponin/1% BSA-PBS buffer with the following panel of antibodies: PE-IL-10, PE-TGF-β, PE-IFN-γ and PE-MHC-II (eBioscience; 1:1000). MNCs were gated using forward and sideward scatter characteristics for lymphocytes and monocytes and at least 10,000 gated events were collected using flow cytometer (BD Biosciences, San Jose, CA, USA). Data were analyzed using the CellQuest software.

### Immunohistochemical Staining

Mice were anesthetized and transcardially perfused with PBS followed by 4% buffered paraformaldehyde. A part of the spleens was removed and frozen in liquid nitrogen. Cryostat sections of the spleens were cut at 40 μm and fixed in acetone for 10 min. For immunohistochemical analysis, nonspecific binding sites were blocked with 2% horse serum (Serotec, Bicester, United Kingdom), and permeabilized with 0.3% Triton in 1% BSA-0.1 M PBS for 60 min. Subsequently, spleen sections were incubated overnight with primary anti-mouse ROCK-II antibody (1:800, BD Bioscience) and 0.1% Triton in 1% BSA-0.1 M PBS. After washing with PBS, reactivity was detected with Alexa Fluor 488 anti-rabbit (1:1000; Molecular Probes Invitrogen, Carlsbad, CA, USA), followed by DAPI (1:1000, Thermo Fisher Scientific) for 30 min, which was used to identify the nuclei. Specificity of DAPI staining was tested by incubating the spleen sections without the primary antibodies. After the immunohistochemistry staining, the spleen sections were covered with 5% glycerol, and examined under a Spinning Disk Confocal Microscope (Cell Observer SD, Carl Zeiss) for immune fluorescence.

### Statistical Analysis

Graphpad Prism 5.0 (Graphpad software, San Diego, CA, USA) was used for statistical analysis. All data were expressed as means ± SEM. Differences between multiple groups were evaluated by the Kruskal-Wallis one-way analysis of variance. Data in the experiments with two groups were tested for statistical significance by using unpaired, two-tailed Student’s *t*-tests. Differences were considered significant if *p* ≤ 0.05.

## Results

### Fasudil Combined With BMSCs Ameliorated Memory Deficits in APP/PS1 Tg Mice

**Figure 1:**
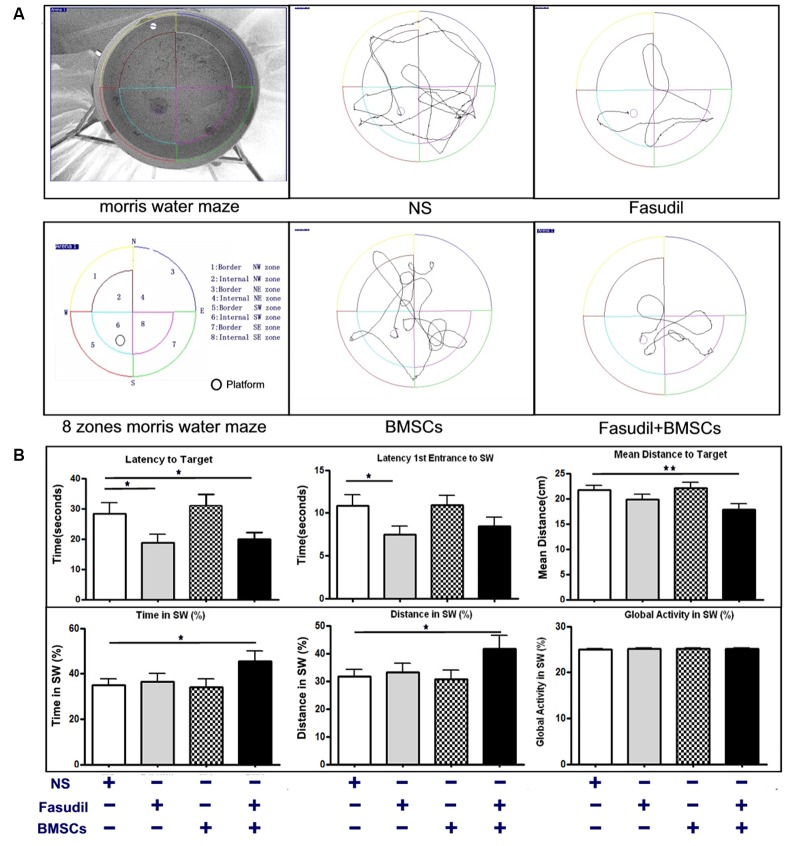
1-(5-isoquinolinesulfonyl)-homo-piperazine (Fasudil) or Fasudil combined with bone marrow stromal cells (BMSCs) improved spatial learning of APPswe/PSEN1dE9 transgenic (APP/PS1 Tg) mice. Mice (8 months old) were intraperitoneally injected with 0.9% saline (NS, *n* = 9), Fasudil (*n* = 8), BMSCs (*n* = 10, tail vein injection), or Fasudil combined with BMSCs (*n* = 10) for 2 months. **(A)** The schematic diagram of the Morris water maze (MWM) test and tracing of movement locus map of each group. **(B)** The corresponding parameters: Latency to Target, Latency 1st Entrance to southwest (SW) zone, Mean Distance to Target, Time in SW (%), and Distance in SW (%), were recorded in the retention test session, representing the time and distance spent by animals from the starting point onto the platform or SW zone. Global activity in SW (%) represents the movement capabilities of each group in SW zone. Data presented are means ± SEM; *p < 0.05, **p < 0.01 as compared to saline (NS).

The MWM test is a commonly used behavioral task for evaluating cognitive deficits (Yu et al., 2017). Efficacy of Fasudil, BMSCs, or Fasudil combined with BMSCs was determined using the MWM test in APP/PS1 Tg mice, which were screened for cognitive impairment (appreciable symptoms) at the age of 8 months and 2 weeks after the last treatment (Figure 1A). Global Activity in SW (%) reflects the body functions of the animal in order to exclude the body dysfunction for swimming. There were no significant differences at baseline among the groups. Latency to the target zone, first-entrance latency to SW, mean distance to the target zone, time in SW, and distance swam in the SW (%) zone were recorded in the retention test session, representing the time taken and distance traveled by animals from the starting point to the platform or SW zone. In the APP/PS1 Tg+Fasudil group, mice showed significantly decreased latency and first-entrance latency to the target zone as compared with APP/PS1 Tg+Saline mice (*p* < 0.05). The APP/PS1 Tg+BMSCs mice did not exhibit statistically significant changes as compared with APP/PS1 Tg+Saline mice in cognitive function. However, the APP/PS1 Tg+Fasudil+BMSCs mice showed significantly decreased latency (*p* < 0.05), first-entrance latency (*p* < 0.05), mean distance traveled to the target zone (*p* < 0.01), and increased time spent (*p* < 0.05) and distance traveled (*p* < 0.05) in the SW zone as compared with APP/PS1 Tg+Saline mice (Figure 1B). Our results demonstrated that Fasudil or Fasudil combined with BMSCs improved spatial learning of APP/PS1 Tg mice, the latter of which appeared to be more effective than Fasudil or BMSCs alone.

### Fasudil Combined With BMSCs Attenuated ROCK-II Expression in the Spleens of APP/PS1 Tg Mice

**Figure 2:**
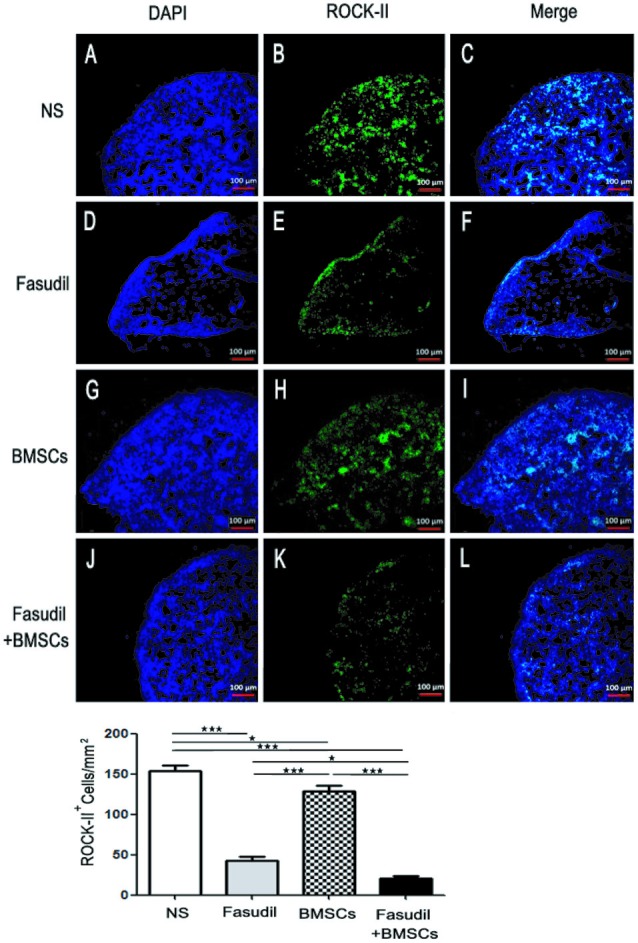
Expression of ROCK II by immunohistochemical staining in the spleens of APP/PS1 Tg mice. Mice were treated with saline (NS), Fasudil, BMSCs, or Fasudil + BMSCs. **(A–C)** The double staining with ROCK II (green) and DAPI (blue) in the spleens of APP/PS1 mice injected with 0.9% saline (×10). **(D–F)** expression of ROCK II inhibited by Fasudil as compared with NS (×10). **(G–I)** expression of ROCK II not significantly inhibited by intravenous injections of BMSCs as compared with NS (×10). **(J–L)** expression of ROCK II significantly inhibited by Fasudil combined with intravenous injections of BMSCs, compared with NS treated mice (×10). Data shown are means ± SEM. *p < 0.05, ***p < 0.001 as compared with saline (NS).

ROCK is expressed both centrally and peripherally. A plethora of studies have demonstrated the role of Rho/ROCK pathway in AD. This pathway is directly involved in the neuronal loss and inhibition of axonal regeneration in AD (Wen et al., 2014). ROCK-II is an important pharmacological target linked to CNS disorders such as AD (Cai et al., 2017). In order to test our hypothesis that expression of ROCK-II was altered by treatments in the peripheral immune system of AD models, we performed immunohistochemical staining in spleens of APP/PS1 Tg mice. As shown in Figure 2, expression of ROCK-II in spleens of APP/PS1 Tg mice was changed by the treatments. More specifically, the double staining of ROCK-II (green) and DAPI (blue) was performed in spleens of APP/PS1 mice treated with 0.9% NS, Fasudil, BMSCs, or Fasudil combined with BMSCs (×10). The expression of ROCK-II was inhibited in APP/PS1 Tg+Fasudil (*p* < 0.001; Figures 2D–F), but not in APP/PS1 Tg+BMSCs mice (*p* < 0.05; Figures 2G–I), as compared with APP/PS1 Tg+NS (Figures 2A–C). In addition, the expression of ROCK-II was significantly inhibited in APP/PS1 Tg+Fasudil+BMSCs (*p* < 0.001; Figures 2J–L) as compared with APP/PS1 Tg+NS, APP/PS1 Tg+Fasudil (*p* < 0.001), or APP/PS1 Tg+BMSCs (*p* < 0.05). Fasudil as an inhibitor of ROCK-II can reduce the expression of ROCK-II in the spleens of APP/PS1 Tg mice; in contrast, BMSCs is not an inhibitor of ROCK-II, thus it potentiated the inhibitory effect of Fasudil on expression of ROCK-II through the immunoregulatory response. Our results suggest an alternative approach for treatment of AD using Fasudil or Fasudil combined with BMSCs through regulation of the peripheral immune system and inhibition of the progression of the disease symptoms.

### Fasudil Combined With BMSCs Affected CD4+ T-Cell Proliferation and Induced the Regulatory T-Cells *in Vivo*

**Figure 3:**
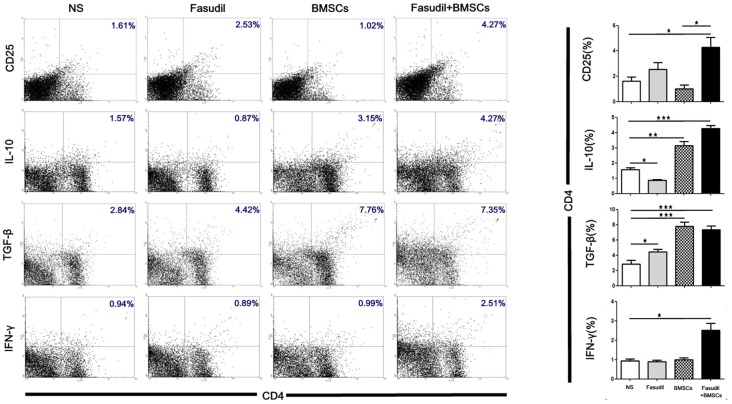
Fasudil, BMSCs, Fasudil combined with BMSCs affected CD4+T-cell proliferation and induced regulatory T cells *in vitro*. Splenic mononuclear cells (MNCs) isolated from APP/PS1 Tg mice were double stained with anti-CD4, anti-CD25, anti-IL-10, anti-TGF-β and anti-IFN-γ antibodies, respectively. An aliquot of 10,000 cells was analyzed using BD flow cytometry. Cells were calculated by quadrant dot plot. Results are expressed as the percentage of double-positive cells. Data were analyzed using two-way analysis of variance (ANOVA) with multiple comparison *post hoc* tests (Bonferroni). Data shown are means ± SEM. *p < 0.05, **p < 0.01, ***p < 0.001 as compared with saline (NS).

Flow cytometry was performed to observe the effects of Fasudil, BMSCs, or Fasudil combined with BMSCs on CD4+ T-cell proliferation and the release of cytokines from T-cells. Splenic MNCs from APP/PS1 Tg mice were isolated and expression of CD25, IL-10, TGF-β and IFN-γ on CD4 T-cells were measured by flow cytometry. All treatment groups were compared with the NS control (Figure 3). In the double staining of CD4+/CD25+, there was no statistically significant difference between Fasudil-treated, BMSCs-treated, or control mice; however, Fasudil combined with BMSCs significantly increased CD4+/CD25+ (*p* < 0.05), suggesting that the combination therapy exhibits more effective regulation of T-cells. Further, the role of IL-10, TGF-β, and IFN-γ was examined by double staining of CD4+ and IL-10+, TGF-β+, or IFN-γ+, respectively. We observed significantly decreased IL-10 (*p* < 0.05) and increased TGF-β (*p* < 0.05) in CD4+ by Fasudil alone, and increased IL-10 (*p* < 0.01) and TGF-β (*p* < 0.05) in CD4+ by BMSCs alone, but no change in IFN-γ by either treatment. In contrast, expression of CD4+/IL-10+ (*p* < 0.001), CD4+/TGF-β+ (*p* < 0.001), CD4+/IFN-γ+ (*p* < 0.05) was significantly increased by Fasudil combined with BMSCs, suggesting greater potency of the combination treatment, which appears to prevent the development of AD pathology and its progression by regulating the CD4+ T-cell proliferation and cytokine release. Our study provides evidence that Tregs can protect the development of AD pathology and its progression. Systemic Tregs administration can ameliorates disease progression and could be an effective AD treatment (Baek et al., 2016).

### Fasudil Combined With BMSCs Shift Macrophages to M2 Phenotype of CD11b and Enhancement the Anti-inflammation

**Figure 4:**
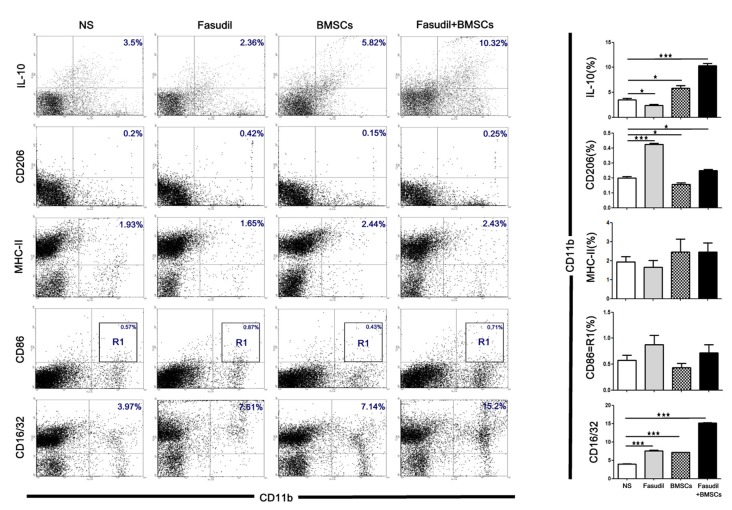
Fasudil or Fasudil combined with BMSCs shifted M1 macrophages to M2 phenotype of CD11b and enhanced the phagocytic ability of CD11b *in vitro*. Splenic MNCs isolated from APP/PS1 Tg mice were stained with macrophage markers for M2 (CD11b+/ IL-10+, CD11b+/ CD206+) or M1 (CD11b+/ MHC-II+, CD11b+/ CD86+), and phagocytic macrophage markers (CD11b+/CD16/32+), and an aliquot of 10,000 cells was analyzed using BD flow cytometry. A part of cells was initially gated on forward and side scatter to remove debris and calculated by quadrant dot plot. Results are expressed as the percentage of double positive cells and shown as means + SEM in each group from a single experiment representative of two independent experiments with similar results. Differences were analyzed using two-way ANOVA with multiple comparison *post hoc* tests (Bonferroni). *p < 0.05, ***p < 0.001 as compared with saline (NS).

We also used flow cytometry to characterize the phenotype of macrophages (M1 and M2 type) after treatment with Fasudil, BMSCs, or Fasudil combined with BMSCs. Macrophages were isolated from splenic MNCs in APP/PS1 Tg mice. Macrophage M2 markers (CD11b+/IL-10+, CD11b+/CD206+), M1 markers (CD11b+/MHC-II+, CD11b+/CD86+), and phagocytic macrophage marker (CD11b+/CD16/32+) were assessed by flow cytometry. All the treated groups were compared with NS (Figure 4). Fasudil, BMSCs, and Fasudil combined with BMSCs significantly increased expression of CD16/32 on CD11b+/CD16/32+ macrophages (*p* < 0.001); combination of Fasudil and BMSCs was more effective than either treatment alone based on the results of expression of the phagocytic macrophage marker. In the double staining of CD11b+/IL-10+, Fasudil significantly decreased expression of IL-10 (*p* < 0.05), while BMSCs significantly increased expression of IL-10 on CD11b+/IL-10+ macrophages (*p* < 0.05); Fasudil combined BMSCs further increased expression of IL-10 on CD11b+/IL-10+ macrophages (*p* < 0.001). In the double staining of CD11b+/CD206+, Fasudil significantly increased the expression of CD206 on CD11b+/CD206+ macrophages (*p* < 0.001). BMSCs and Fasudil combined with BMSCs also significantly increased the expression of CD206 on CD11b+/CD206+ macrophages (*p* < 0.05). In contrast, in the double staining of CD11b+/MHC-II+ and CD11b+/CD86+, there were no significant changes in the expression of MHC-II or CD86 between the treatment group of Fasudil, BMSCs, or Fasudil combined with BMSCs and the NS control. The expression of macrophage M1 markers was not significantly affected by the treatments, but the combination of Fasudil and BMSCs significantly increased the expression of macrophage M2 markers. The results suggest that Fasudil or Fasudil combined with BMSCs shifts M1 (MHC-II, CD86) to M2 (IL-10, CD206) phenotype of CD11b, significantly enhancing the anti-inflammatory and phagocytic ability (CD16/32) of CD11b macrophages.

## Discussion

We previously demonstrated that Fasudil reduced Aβ deposition and ROCK-II expression in the brain of APP/PS1 Tg mice (Gu and Zhang, 2017; Yu et al., 2017). In the present study, we further showed that Fasudil or BMSCs used alone was effective to decrease the severity of AD-related symptoms through peripheral immune system responses, however, the combination of both showed greater efficacy.

The main symptom of AD is cognition deficit, which is considered to be caused by excessive formation of Aβ-protein (Selkoe, 1999). One of the most promising approaches toward the Aβ clearance is the use of immunotherapies (Jesudason et al., 2007). Although brain pathology and relative changes have been well studied, their impact on inflammatory processes in the peripheral immune system, including the spleen, have not been investigated (Subramanian et al., 2010). Abnormal activation of ROCK has been found in animal models of AD. ROCK activation increases the production of Aβ in the brain. Therefore, we hypothesized that ROCK-II was a potential drug target for the treatment of AD. Recent studies have shown that ROCK-II inhibitors protect neurons by inhibiting caspase-3 (Xiao, 2008), leading to increases in the number of axons in hippocampal neurons. Inhibition of ROCK-II also reduces Aβ1–42 levels in the nervous system (Aleksis et al., 2017). In the MWM test, improvement in spatial learning of APP/PS1 Tg mice was observed following treatment with Fasudil or Fasudil combined with BMSCs; the combination therapy was even more effective. BMSCs, which are not ROCK-II inhibitors, potentiated the effect of Fasudil, a ROCK-II inhibitor, in terms of reduction of ROCK-II expression. This is probably due to the immunoregulatory ability of BMSCs. Decreased expression of ROCK-II in the spleen produced by the combination of Fasudil and BMSCs appears to be associated with enhanced immunity in the brain, or at least resulted from bidirectional immunity communication between the brain and spleen. A cascade of event will follow to reduce Aβ or reduce the phosphorylation of tau.

In the peripheral immune system, Fasudil combined with BMSCs significantly increased CD4 T-cell proliferation and cytokine release and regulated the immune system to work functionally. TGF-β1 has been shown to suppress glial and T-cell mediated neuroinflammation; its inhibitory effect on Aβ neurotoxicity has been considered as a potential therapeutic approach in AD patients (Chen et al., 2015). This phenomenon appears to be linked to a switch mechanism from a Th1 effector to an IL-10-mediated regulatory response (Loewenbrueck et al., 2010); release of IFN-γ from infiltrating Th1 cells accelerates markers of disease in an animal model of AD (Browne et al., 2013). Tregs have an impact on cognitive function. Inflammatory conditions defined as excessive activation of immune cells and release of cytokines, are associated with bidirectional immune system-brain communication (Rosas-Ballina et al., 2015). The cytokines of T-cell production have the ability to increase macrophage phagocytosis. Cytokine production is a key pathologic event in pro-inflammatory and anti-inflammatory responses in AD (Gu and Zhang, 2017).

We further explored the role of the immune system and the possible mechanism involved. It was found that Fasudil combined with BMSCs switched M1 (MHC-II, CD86; Rosas-Ballina et al., 2015) to M2 (IL-10, CD206; Rosas-Ballina et al., 2015) phenotype of CD11b, significantly increasing anti-inflammatory cytokines and enhancing the phagocytic ability of macrophages (CD 16/32; Makarenkova et al., 2006). Our data showed a trend of increased CD86 and MHC (M1 markers), but it was not statistically significant, indicating a major effect on M2, instead of M1, which may be caused by the transformation from M1 to M2; leading to increased production of the M2 regulatory cytokine IL-10 (Bispo da Silva et al., 2017). We observed decreased IL-10 and increased CD206 expression after Fasudil treatment. In contrast, both were significantly increased by the combination treatment, suggesting the progression of the disease. AD is a chronic disease and inflammation forms gradually. Our results revealed that the resting phase of M1 (pro-inflammatory) started to be activated as indicated by increased pro-inflammatory factors, and then turned into M2 phenotype exhibiting anti-inflammation, as shown by increased CD206, an M2 marker. Tregs are regulators that increase the ability of macrophages to devour inflammation. T lymphocytes have to pass through two main barriers: the blood-cerebrospinal fluid barrier (BCSFB) and the blood brain barrier (BBB). In our previous studies, we have demonstrated the protective effects of Fasudil on BBB function in EAE mice. Fasudil may produce inhibition of the down-regulation of Occludin and Zo-1 in the brain, indicating that Fasudil maintains the integrity of BBB (Gu et al., 2017). All types of peripheral immune cells may infiltrate through the pathologically altered BBB during AD pathogenesis (Mietelska-Porowska and Wojda, 2017).

In summary, our study demonstrated that Fasudil, a ROCK-II inhibitor, alone or in combination with BMSCs, improved cognitive function of APP/PS1 Tg mice through the regulation of the peripheral immune system, including decreased expression of ROCK-II, induction of Tregs, switching pro-inflammatory M1 to anti-inflammatory M2 phenotype, and increasing phagocytic macrophages in the spleen. The immunological reaction in the CNS was decreased through inhibition of the peripheral immunity after treatment with Fasudil in combination of BMSCs, leading to improvement of CNS microenvironment and promoting cognitive function. Further studies are required for the detailed mechanisms of interaction of immunity between the CNS and the peripheral immune system.

## Author Contributions

JY and YY designed the study, carried out the mouse behavioral tests, immunoassays and flow cytometry. C-GM and YJ conceived the study, participated in its design and coordination and helped draft the manuscript. B-GX, H-TZ, G-XZ, GK and JC participated in its design and revised the manuscript. H-TZ revised and finalized the manuscript. QG, YZ and HY performed the immunoassays and flow cytometry experiments. CL, YG and ZC participated in the statistical analysis. All authors read and approved the final manuscript.

## Conflict of Interest Statement

The authors declare that the research was conducted in the absence of any commercial or financial relationships that could be construed as a potential conflict of interest.
